# Comparison of snack characteristics by diet quality findings from a nationally representative study of Australian adolescents

**DOI:** 10.1038/s41598-024-75386-1

**Published:** 2024-10-10

**Authors:** Binyam Girma Sisay, Kathleen E. Lacy, Sarah A. McNaughton, Rebecca M. Leech

**Affiliations:** 1 Institute for Physical Activity and Nutrition, School of Exercise and Nutrition Sciences, Deakin University, Burwood Campus, 221 Burwood Highway, 3125 Burwood, VIC Australia; 2 Health and Well-Being Centre for Research Innovation, School of Human Movement and Nutrition Sciences, University of Queensland, St Lucia, QLD 4067 Australia

**Keywords:** Snacks, Adolescents, Energy density, Diet quality, Nutrition, Public health

## Abstract

Snacking is a common dietary behaviour among adolescents contributing to more than one quarter of their total energy intake; however, the relationship between snacks and diet quality remains unclear. Hence, this study aimed to examine the characteristics of snacks among adolescents with different levels of diet quality. Dietary data collected from a nationally representative sample of Australian adolescents (12–18 years old, *n* = 935) using one 24-hour dietary recall in the National Nutrition and Physical Activity Survey were analysed. Snacks were defined based on participant-identified eating occasions, and diet quality was assessed using the Dietary Guideline Index for Children and Adolescents (DGI-CA). Marginal means and proportion of snack characteristics including snack frequency, snack energy density (ED), and commonly consumed foods at snack from the five food groups and discretionary foods at snack across DGI-CA tertiles (highest tertile indicating high guideline adherence) were estimated for both boys and girls using linear regression and logistic regression. Differences in means were tested using the F-test. The results showed no significant differences in the mean frequency of snacks across tertiles of DGI-CA scores. The mean ED of snacks decreased as DGI-CA scores increased in both boys (lowest tertile = 8.4, 95% CI [7.1, 10.0] kJ/g, highest tertile = 6.3 [5.4, 7.4] kJ/g) and girls (lowest tertile = 9.0 [7.8, 10.3] kJ/g, highest tertile = 5.9 [5.1, 6.9] kJ/g). As diet quality improved, the proportion of adolescents consuming discretionary (i.e., unhealthy) foods and foods from the five food group foods as snacks decreased and increased, respectively. In conclusion, adolescents with higher diet quality consumed snacks with a lower ED while lower proportion of them consume discretionary foods, and higher proportion of them consume from the five food groups. Encouraging the consumption of foods from the five food groups with lower ED as snacks presents an opportunity to enhance adolescent diet quality.

**Supplementary Information:**

The online version contains supplementary material available at 10.1038/s41598-024-75386-1.

## Introduction

Poor diet quality is prevalent among adolescents^1,2^. It is characterized by high consumption of discretionary foods (i.e., energy-dense and nutrient-poor foods and beverages), refined grains, and low intake of fruits and vegetables^2,3^. Poor diet quality is one of the factors that has been linked to diet-related health outcomes including obesity^4^. In 2020, 10% of boys and 8% of girls between the ages of 5 and 19 years globally were living with obesity^5^. It has been suggested that dietary behaviours formed during adolescence may persist into adulthood^6,7^. Therefore, it is imperative to prioritize research into the dietary behaviours that contribute to healthier eating patterns in adolescents^8^.

Snacking is a common, yet not well understood, dietary behaviour among adolescents. Snacking is an ambiguous term that can refer to different behaviours, including consuming foods that are rich in energy but poor in nutrients, or eating between meals^9,10^. Snacking has been examined in relation to the number of snacks consumed (frequency per day), the energy density (ED) of snacks and the types of foods consumed as snacks^11–15^. Snacking is highly prevalent among adolescents, contributing 7.9 to 30.5% of their daily energy intake, and nearly one quarter of their daily energy fat, saturated fat and total sugar intake^10,16,17^. Despite the high prevalence of snacking and its significant contribution to energy intake, there is a lack of understanding regarding the relationship between snacking and diet quality due to the conflicting findings reported across a limited number of studies.

Most studies examining snacking among adolescents have focused on its frequency^11,18–20^. A study of U.S. children and adolescents found the relationship between snack frequency and diet quality varied according to the approach used to define snacks^18,19^. Snack frequency defined by the energy contribution of eating occasions (EO) to overall energy intake (EI) (EO contributing < 15% of total EI was considered a snack) was positively associated with diet quality. However, in the same study, no associations were found when snacks were defined based on participant self-reported EO as a snack or the time of EO consumption (EO that fell outside of this time intervals 05:30–9:30, 11:30–14:30 and 17:00–21:30 h were identified as snacks^21^)^18^. In contrast, a study conducted among British children and adolescents reported that snack frequency was inversely associated with the Mediterranean Diet Score (MDS), an indicator of diet quality, when snacks were defined based on the time of day and their contribution to overall EI^11^. Further, another study involving British adolescents found a positive association between snack frequency and the Diet Quality Index for Adolescents (DQI-A). However, an inverse association emerged when EOs containing fewer than 50 kcal (209 kJ) were omitted^20^. This might be explained by the exclusion of EO which may contain small amounts of foods and/or beverages with lower ED, such as low-energy beverages, fruits, and vegetables, which could have made a positive contribution to overall diet quality.

The conflicting findings regarding snacks and diet quality may be due to the varied approaches used to define snacks across studies and the examination of snack frequency, which is a simple measure that does not reflect the nutritional quality of snacks. One study in British adolescents assessed ED of snacks^13^ and found a weak inverse association between snack ED and MDS. Therefore, snack ED may be a better indicator of the nutritional quality of snacks. For example, in a study by Murakami and Livingstone^13^, lower ED snacks were linked to the consumption of vegetables, fruits, and dietary fibre, contributing to a higher overall diet quality. However, there is a lack of evidence on the variation in foods consumed at snacks among those with high and low diet quality.

Overall, there is limited evidence on the contribution of snacks to overall diet quality, with the majority of studies examining snack frequency. Examination of multiple snack characteristics (snack frequency and snack ED) and foods commonly consumed at snacks among adolescents with different levels of diet quality (i.e., low diet quality versus high diet quality) may provide a new perspective on the role of snacks in adolescent diet quality. Hence, the aim of this study is to examine the characteristics of snacks, including common food choices at snacks, according to different levels of diet quality, among a nationally representative sample of Australian adolescents aged 12–18 years. The findings of this study could be used to develop public health strategies and messages specifically tailored to promote the consumption of foods at snacks that have been linked to better diet quality among adolescents.

## Methods

### Sample and study design

The present cross-sectional study utilized data from the 2011–2012 National Nutrition and Physical Activity Survey (NNPAS), which was conducted nationwide by the Australian Bureau of Statistics (ABS) from May 2011 to June 2012. The survey employed a multistage stratified area probability sampling design to recruit participants. Detailed descriptions of the survey’s rationale, design, and methods can be found in previous literature^22^. In summary, the survey included private households across all Australian states and territories, including both urban and rural areas. For each participating household, one adult (aged 18 years or older and a regular resident) and, if applicable, one child aged 2–17 years were randomly selected. Sociodemographic, dietary, and anthropometric information were collected from participants during an in-person interview^22^.

The final sample comprised 9,519 households, representing a household response rate of 77%. In total, 12,153 participants were selected from these households. Among them, 1,101 were adolescents between the ages of 12 and 18 years. Adolescents aged between 12 and 18 years were selected to align the findings of our study with the 2013 Australian Dietary Guidelines, which provide dietary recommendations for adolescents within a similar age range^23^.

The Census and Statistics Act 1905 provides ethics approval for the ABS to conduct the household interview components of health surveys^24^. As this study involves the secondary analysis of pre-existing and nonidentifiable data, an exemption from ethics review was approved by the Deakin University Human Research Ethics Committee ([DUHREC]; application 2023 − 193).

### Dietary assessment

Dietary data were collected by administering two 24-hour recalls, utilizing an adapted U.S. Department of Agriculture (USDA) automated multiple-pass method^25,26^. The dietary recall interviews were conducted by trained ABS staff. Dietary assessments were conducted across all seasons, from May 2011 to June 2012. The initial recall was conducted through face-to-face interviews with ABS personnel who had undergone specialized training. The subsequent recall was carried out via telephone after at least eight days but was limited to only 60% among those approached. Adolescents aged 12–14 years were interviewed directly in the presence of a proxy, whereas those aged 15–17 years were interviewed directly after obtaining informed consent from their parent or guardian. Adolescents aged 18–19 years were interviewed directly^22^. The Australian Supplement and Nutrient Database 2011–2013 was used to calculate energy intake (kJ) and, nutrient intake (g) from all foods and drinks reported during the recalls^22^. To maximize adolescent participation and maintain the national representativeness of the sample, dietary information collected on the first day of recall was used for this analysis. Foods commonly consumed during snacks were identified using the Food Standards Australia New Zealand (FSANZ) 8-digit food code, determined by their frequency of consumption during snacks. Food and beverage intakes of the five food group foods and discretionary foods (g/d) were calculated using the Australian Dietary Guidelines (ADG) database^23^ developed for the NNPAS 2011–12. For the ADG Database, the ABS disaggregated and estimated all individual food components for the five food group foods within mixed dishes. In the database, discretionary food and beverage items and mixed dishes were flagged by the ABS.

### Definition of snacks

During the dietary recall, adolescents reported the start time of each eating occasion (EO) and selected the type of EO that occurred in the previous 24 h. The available response options included breakfast, brunch, lunch, dinner, supper, snack, morning tea, afternoon tea, drink/beverage, extended consumption, and others. Any EO that was self-reported as “breakfast”, “brunch”, “lunch”, “dinner”, or “supper” was considered a meal. While all other self-reported EOs, including “snacks”, “morning/afternoon tea”, “beverage/drink”, “extended consumption” and “other” were classified as snacks. Consistent with previous literature^21,27^, EOs labelled by adolescents as “extended consumption” or “other/don’t know” were categorised as meals or snacks if they occurred concurrently or within 15 min of the EO window that the participant identified as a meal or snack. Any remaining extended consumption occasions were then coded as snacks^27^. EOs with less than 210 kJ were excluded, and any snacks that occurred within 15 min of each other were combined and treated as one snack^18,21^. This participant-identified approach to defining EO with additional criteria (15-minute time interval, along with the 210-kJ criterion) has been shown to be the best predictor of variance in total energy intake among Australian children and adolescents. Moreover, there was good agreement for snack frequency when using the time of the day vs. participant-identified approach (intraclass correlation coefficient 0.93)^21^.

### Snack characteristics

The mean snack frequency per day was estimated using the participant-identified snack definition (as described above). Two different energy density (ED) calculations were used: one included all food and beverages, and the other excluded beverages. The approach implemented for the calculation of ED, excluding beverages, followed the method previously described by Vernarelli, et al.^28^. Beverages containing or not containing energy were excluded. However, tap water and milk (including non-dairy milk substitutes) were treated differently when consumed as part of food because these beverages are often consumed as ingredients or additional components in other food items (e.g., milk added to ready-to-eat cereals or tap water added to cooked cereals). The ED of snacks was calculated by first determining the energy and weight from snacks, including, and excluding beverages, respectively. Then, the ED of snacks with and without beverages was calculated by dividing the total energy content from snacks by their corresponding total weights. Snacks were further classified into two major groups: those belonging to the five food groups and those belonging to discretionary foods, in line with Australian Dietary Guidelines. The five food groups consisted of1) vegetables and legumes/beans; 2) fruit; 3) grain (cereal) foods, primarily wholegrain and/or high cereal fibre varieties; 4) lean meats, poultry, fish, eggs, tofu, nuts and seeds, and legumes/beans; and 5) milk, yoghurt, cheese, and/or alternatives, mainly reduced-fat options^23^. In contrast, discretionary foods include choices that are high in energy, saturated fat, added sugars, and/or salt, or those containing alcohol^23^.

### Assessment of diet quality

The Dietary Guideline Index for Children and Adolescents (DGI-CA) was used to measure diet quality^29^. The food group and nutrient components, as well as the development and evaluation of DGI-CA, have been described in detail elsewhere^29^. Briefly, the DGI-CA reflects the adherence of children and adolescents to the 2013 Australian Dietary Guidelines. DGI-CA includes nine components, of which seven assess adequate intake of recommended foods and beverages, each worth a maximum score of 10 points:1) a variety of nutritious foods; 2) vegetables including legumes/beans; 3) grains mostly whole grain and/or high fibre; 4) fruit; 5) lean meat and poultry, fish, eggs, tofu, nuts/seeds, and legumes/beans; 6) dairy and/or alternatives mostly reduced fat; and 7) drinking plenty of water. The remaining two components assess foods and beverages where limited intake is encouraged:8) limit intake of saturated fat, alcohol, and added salt and sugars (worth 20 points) and 9) replace saturated fats with unsaturated fats (worth 10 points). The highest and lowest scores for each indicator were established based on age- and sex-specific serving recommendations. Partially meeting the recommendations resulted in the assignment of proportional scores. The scores for the nine components were summed to obtain a total score, with a maximum of 100 points. A higher score indicates higher diet quality. The DGI-CA total score was divided into tertiles for further analyses. Adolescents in the top tertile (3rd tertile) were considered to have higher diet quality, whereas those in the bottom tertiles (1st and 2nd tertiles) were considered to have lower diet quality.

### Sociodemographic characteristics

Age (years) and sex (male/female) were reported by the participants. The participants’ household postal codes were used to assign the socioeconomic index for areas (SEIFA), which indicates the area level of disadvantage. The SEIFA scores range from one (indicating the most disadvantaged) to five (representing the most advantaged)^21,22^.

### Compliance with physical activity guidelines

The level of physical activity reported by adolescents aged 12 to 18 years was assessed using a self-reported questionnaire. Adolescents between the ages of 12 and 17 years reported the time spent in different types of moderate-to-vigorous physical activities and active transport during the previous 7 days. The reported time spent on each of these activities was added together. In addition, these adolescents reported the time spent in the past 7 days sitting or lying down to watch TV or DVD, playing any electronic game and using internet or computer for homework and non-homework purposes. Those who spent at least 60 min per day on moderate-to-vigorous physical activity and no more than two hours screen-based activity for entertainment/non-educational purposes a day were considered to have met the physical activity recommendations^30^. Older (18 years) adolescents’ physical activity levels were assessed using the Active Australia Survey^31^. Older adolescents reported the frequency and time spent in different types of moderate/vigorous physical activities, vigorous gardening, strength and toning activities, and active transport. The reported duration of each activity was summed to estimate the total time spent on moderate and vigorous physical activity. Those who engaged in physical activity for at least 150 min over five or more sessions per week were considered to have met the physical activity recommendations^31^.

### Evaluation of potential energy intake misreporting

To evaluate potential energy misreporting, the method outlined by Huang, et al.^32^, the ratio of energy intake (EI) to estimated energy requirement (EER) was used. The EER was calculated using established Dietary Reference Intake (DRI) equations that consider specific characteristics such as sex, age, body weight, height, and level of physical activity^33^. Due to the lack of objective physical activity data a “low active” level of physical activity (≥ 1.4 to < 1.6) was assumed for all adolescents.

Height and weight used in the EER calculation was collected voluntarily, using a standard procedure. To measure weight, digital scales with a maximum capacity of 150 kg were used, whereas height was measured using a stadiometer capable of measuring up to 210 cm. Weight was recorded in kilograms with one decimal place, whereas height was recorded in centimetres with one decimal place. To ensure accuracy, 10% of participants were randomly selected for additional height measurements. If there was a difference of more than one centimetre between the first and the second height measurements, a third measurement was taken. Although, weight measurements were only recorded once, regularly calibrated digital scales and standard procedures were used to minimize measurement error^24^.

### Analytic sample

The present study included adolescents aged 12–18 years who completed the first 24-hour dietary recall (*n* = 1101; Supplementary File 1). Pregnant or breastfeeding individuals (*n* = 2) and those who had engaged in shift work in the past four weeks (e.g., night or rotating shifts affecting eating patterns) were excluded (78 adolescents aged 14–18 years). A total of 1021 adolescents were initially eligible for inclusion in this study. Participants were further excluded if they did not report the time of eating (*n* = 2), or if they did not report having any snacks (*n* = 84 excluded). The final analysis included 935 participants (487 boys and 448 girls).

### Statistical analysis

All statistical analyses were performed using Stata 17 (Stata Corp LP), and the NNPAS person and replicated weights were applied to adjust for the probability of selection, nonresponse, and multistage survey design. Descriptive statistics were estimated for the full sample and stratified by sex. Several studies have indicated that snack characteristics, particularly snack frequency, vary by sex^15,16,18,34^. To examine differences in snack characteristics between sexes, as well as variations in the association between snack characteristics and diet quality based on sex, all statistical analyses were stratified by sex. Weighted proportions with corresponding 95% confidence intervals (CI) were used for categorical variables, whereas weighted means with corresponding 95% confidence intervals were used for continuous variables. Differences in area-level disadvantage (SEIFA) quintiles between boys and girls were tested using the adjusted Wald test (TEST command).

Multivariate linear regression models, stratified by sex, were employed to ascertain the adjusted means and 95% confidence interval (CI) of snack characteristics (snack frequency and ED) by tertiles of diet quality scores. The models were adjusted for age (continuous), area-level disadvantage (SEIFA; categorical), and EI: EER (continuous; to adjust for potential energy intake misreporting). To ensure that the models met the assumptions for linear regression, the following variables were log-transformed and included: snack frequency, energy density (ED) of snacks including beverages (kJ/g), ED of snacks without beverages (kJ/g), weight of snacks including beverages (g), and weight of snacks excluding beverages (g). The F-test was used to test the differences between marginal means across tertiles of diet quality. Multivariate logistic regression, stratified by sex, was employed to determine the adjusted proportion of adolescents who consumed the top five most consumed five food group foods and discretionary foods as a snack, respectively, at different levels of diet quality, along with their corresponding 95% CI. The models were adjusted for age, area-level disadvantage (SEIFA), and EI: EER (to adjust for energy misreporting). The chi-square test was used to test the differences between the marginal proportions across tertiles of diet quality. P values less than 0.05 were considered statistically significant. Means and proportions with non-overlapping CI are significantly different form one another (*p* < 0.05).

To explore the difference in sex and tertile-specific commonly consumed foods at snacks, we also calculated the proportion of adolescents who consumed the top five snacks within each tertile of diet quality stratified by sex. Additionally, when examining how milk is consumed as a beverage or in combination with other foods, we determined the proportions of adolescents who consumed milk as a beverage with additions, or milk as an addition with cereal, stratified by sex.

As a sensitivity analysis, the impact of excluding EOs with low energy contribution (210 kJ or less) on the distribution of snack characteristics across three tertiles of DGI-CA was explored. The marginal mean of snack characteristics across tertiles of DGI-CA were determined using the same approach as described above. The top five commonly consumed foods at snack were also identified for all EOs, including those with an energy contribution below 210 kJ, for both boys and girls.

## Results

Table 1 presents the characteristics of adolescent participants in the NNPAS. No differences in the mean or proportion of age, DGI-CA scores, meets physical activity guidelines, Area-level disadvantage (SEIFA) except for the lowest 20% were observed between boys and girls.

**Table 1:** Characteristics of adolescent participants (12–18 years) in the National Nutrition and Physical Activity Survey 2011-12.

Characteristics	Boy (*n* = 487)		Girl (*n* = 448)		*P* value^1^
	Mean	95% CI	Mean	95% CI	
Age (Years)	14.7	14.5, 15.0	14.6	14.3, 14.9	0.49
Area-level disadvantage (SEIFA)^2^ quintiles. (%)					
Lowest 20%	18.4	13.9, 24.0	12.7	8.3, 19.1	0.08
Second quintile	20.0	15.1, 26.1	19.0	14.2, 25.0	0.77
Third quintile	18.6	13.3, 25.3	26.4	18.9, 35.0	0.12
Fourth quintile	18.7	14.2, 24.2	19.1	14.2, 25.1	0.92
Highest 20%	24.3	18.3, 31.6	22.8	17.8, 28.6	0.68
Meets physical activity guidelines (%)	11.0	7.4, 16.2	7.0	4.5, 10.8	0.15
DGI-CA score	45.1	43.4, 46.7	44.0	45.7, 46.7	0.45
DGI-CA, Dietary Guideline Index for Children and Adolescents ^29^ Results are presented as weighted percentages (%) or weighted means (95% CI) ^1^ P-value for differences between boys and girls based on F test (continuous variable) or adjusted Wald test (categorical variables) ^2^Australian Bureau of Statistics Socio-Economic Indexes for Areas. SEIFA quintiles range from one (most disadvantaged) to five (most advantaged)					

Tables 2 and 3 show the characteristics of snacks across the DGI-CA categories among adolescent boys and girls aged 12–18 year from the NNPAS. There were no observed differences in the mean frequency of snacks among adolescents across tertiles of DGI-CA scores, regardless of sex. The mean ED of snacks, both with and without beverages, decreased as DGI-CA scores increased from the lowest tertile to the highest tertile. Additionally, the mean differences in ED for snacks without beverages across tertiles of DGI-CA scores were statistically significant in both boys and girls (p = < 0.05). However, the mean differences in ED for snacks with beverages across tertiles of DGI-CA scores were statistically significant among girls (p = < 0.05) but not for boys.

**Table 2:** Characteristics of snacks across DGI-CA categories among adolescent boys aged 12–18 year from the National Nutrition and Physical Activity Survey 2011 − 12.^1^.

			Tertile of Diet Quality (DGI-CA)						
	All		1st tertile (*n* = 160)		2nd tertile (*n* = 146)		3rd tertile (*n* = 181)		P value^2^
Characteristics	Mean	95% CI	Mean	95% CI	Mean	95% CI	Mean	95% CI	
Snack frequency	2.2	2.2, 2.3	2.2	2, 2.4	2.3	2, 2.6	2.1	1.9, 2.3	0.63
ED from snacks									
ED of snacks including beverages (kJ/g)	5.5	5.0, 6.1	5.9	5.1, 6.9	5.8	4.8, 6.9	5.9	4.3, 5.7	0.14
ED snacks without beverages (kJ/g)	7.4	6.7, 8.2	8.4	7.1, 10.0	7.7	6.6, 9.0	6.3	5.4, 7.4	**0.03**
EI from snacks									
EI including beverages (kJ)	3112	2851, 3372	3279	2886, 3672	3367	2659, 4075	2801	2482, 3120	0.07
EI without beverages (kJ)	2806	2569, 3044	2911	2493, 3329	3021	2396, 3647	2596	2271, 2920	0.27
Weight of food consumed from snacks									
Weight including beverages (g)	419.9	372.2, 473.6	433.6	360, 522.3	423.9	324.9, 553.1	416.8	360.8, 481.5	0.94
Weight excluding beverages (g)	277.3	246.7, 311.6	255.5	201.9, 323.3	279.2	221.1, 352.5	306.7	255.9, 367.5	0.43
DGI-CA, Dietary Guideline Index for Children and Adolescents ^29^ ED, energy density EI, energy intake ^1^Results are presented as weighted means (95% CI), estimates were determined using multivariate liner regression (adjusted mean), multivariate logistic regression (adjusted proportion), adjusted for age, area-level disadvantage (SEIFA) and reported energy intake to estimated energy requirement (EI: EER). Means with non-overlapping CI are significantly different form one another (*p* < 0.05) ^2^ P-value for trend of differences across tertiles based on F test (continuous variable)									

**Table 3:** Characteristics of snacks across DGI-CA categories among adolescent girls aged 12–18 year from the National Nutrition and Physical Activity Survey 2011-12.

			Tertile of Diet quality (DGI-CA)						
	All		1st tertile (*n* = 153)		2nd tertile (*n* = 151)		3rd tertile (*n* = 144)		P value^1^
Characteristics	Mean	95% CI	Mean	95% CI	Mean	95% CI	Mean	95% CI	
Snack frequency	2.0	1.9, 2.1	1.9	1.7, 2.1	1.9	1.7, 2.2	2.2	1.9, 2.4	0.29
ED from snacks									
ED of snacks including beverages (kJ/g)	6.0	5.6, 6.5	6.8	5.9, 7.7	6.3	5.3, 7.4	5.3	4.6, 6.0	**0.0427**
ED snacks without beverages (kJ/g)	7.3	6.7, 7.9	9.0	7.8, 10.3	7.30	6.2, 8.6	5.9	5.1, 6.9	**< 0.001**
EI from snacks									
EI including beverages (kJ)	2617	2403, 2830	2967	2644, 3290	2563	2119, 3007	2253	1917, 2588	**0.007**
EI without beverages (kJ)	2436	2227, 2645	2767	2440, 3093	2509	2076, 2942	1968	1657, 2279	**0.007**
Weight consumed from snacks									
Weight including beverages (g)	328.9	292.9, 369.4	333.2	275.2, 403.3	308.5	237.3, 401.1	336.6	276.9, 409.2	0.87
Weight excluding beverages (g)	251.8	224.0,283.0	237.3	190.4, 295.7	258.0	201.8, 329.7	251.7	207.5, 305.4	0.88
DGI-CA, Dietary Guideline Index for Children and Adolescents ^29^ ED, energy density EI, energy intake Results are presented as weighted means (95% CI) ^1^ Results are presented as weighted means (95% CI), estimates were determined using multivariate liner regression (adjusted mean), multivariate logistic regression (adjusted proportion), adjusted for age, area-level disadvantage (SEIFA) and reported energy intake to estimated energy requirement (EI: EER). Means with non-overlapping CI are significantly different form one another (*p* < 0.05)									

Tables 4 and 5 show the proportion of boys and girls consuming commonly consumed foods as snacks by DGI-CA tertiles. Boys and girls with higher diet quality (3rd tertile of DGI-CA scores) were more likely to consume foods from the five food groups at snacks except for full fat milk, than those with lower diet quality (1st and 2nd tertiles). In boys, the difference in the proportion of those consuming fruit juices (commercially prepared) (*P* = 0.02), apples (*P* < 0.001), bananas (*P* < 0.001), and regular whole milk (*P* = 0.04) varied across tertiles of DGI-CA. However, among girls, the difference in the proportion of those consuming commonly consumed snacks was only statistically significant for apples (*P* < 0.001) and bananas (*P* = 0.01) across tertiles of DGI-CA.

**Table 4:** Proportion of boys (aged 12–18 years) consuming commonly consumed foods as snacks by DGI-CA tertiles - National Nutrition and Physical Activity Survey 2011-12.

	Tertile of Diet quality (DGI-CA)								
	All		1st tertile (*n* = 160)		2nd tertile (*n* = 146)		3rd tertile (*n* = 181)		P value^1^
Characteristics	%	95% CI	%	95% CI	%	95% CI	%	95% CI	
Foods from the five food groups commonly consumed as snacks (%)									
Apples	22.2	17.1, 28.3	7.3	2.9, 11.6	25.0	17.4, 32.6	29.9	22.5, 37.2	**< 0.001**
Milk, cow, fluid, regular whole, full fat	14.2	9.8, 20.2	10.4	5.3, 15.5	20.8	13.7, 28.0	10.5	5.7, 15.3	**0.04**
Bananas	8.3	5.1, 13.4	0.7	0.7, 2.2	8.0	3.2, 12.7	13.7	8.3, 19.1	**< 0.001**
Breads, and bread rolls, white, mandatorily fortified	6.5	3.7, 11.1	5.9	2.0, 9.8	5.6	1.5, 9.7	6.6	2.7, 10.4	0.94
Fruit juices, commercially prepared	5.9	3.2, 10.7	1.4	0.5, 3.4	5.0	1.1, 8.9	7.2	3.1, 11.3	**0.02**
Discretionary foods commonly consumed as snacks (%)									
Potato crisps	16.3	12.6, 20.8	16.0	9.9, 22.1	19.7	12.9, 26.6	11.9	6.7, 17.2	0.20
Soft drinks, cola	12.2	8.6, 16.9	19.7	13.1, 26.2	7.2	2.6, 11.7	7.9	3.6, 12.2	**0.005**
Savoury biscuits, wheat based, plain, energy > 1800 kJ per 100 g	9.1	6.1, 13.3	10.7	5.5, 15.9	10.5	5.3, 15.7	5.4	1.8, 9.0	0.14
Soft drinks, non-cola	8.7	5.7, 13.1	13.4	7.7, 19.1	8.5	3.4, 13.6	5.2	1.7, 8.7	0.05
Chocolate (plain, unfilled varieties)	6.8	4.3, 10.7	8.3	3.8, 12.7	7.9	3.4, 12.4	2.2	0.2, 4.6	**0.01**
DGI-CA, Dietary Guideline Index for Children and Adolescents ^29^ ED, energy density Results are presented as weighted proportion (95% CI) ^1^ P-value for differences across tertiles based on Chi-squared test Results are presented as weighted means (95% CI), estimates were determined using multivariate liner regression (adjusted mean), multivariate logistic regression (adjusted proportion), adjusted for age, area-level disadvantage (SEIFA) and reported energy intake to estimated energy requirement (EI: EER). Means and proportions with non-overlapping CI are significantly different form one another (*p* < 0.05).									

**Table 5:** Proportion of girls (aged 12–18 years) consuming commonly consumed foods as snacks by DGI-CA tertiles - National Nutrition and Physical Activity Survey 2011-12.

			Tertile of Diet quality (DGI-CA)						
	All		1st tertile (*n* = 153)		2nd tertile (*n* = 151)		3rd tertile (*n* = 144)		P value
Characteristics	%	95% CI	%	95% CI	%	95% CI	%	95% CI	
Foods from the five food groups commonly consumed as snacks (%)									
Apples	16.7	12.1, 22.6	11.1	5.6, 16.6	9.6	4.6, 14.7	28.9	20.6, 37.2	**0.001**
Milk, cow, fluid, regular whole, full fat	7.3	4.9, 10.7	10.2	4.9, 15.4	7.5	3.0, 12.1	4.5	0.7, 8.4	0.22
Bananas	7.1	4.1, 12.1	2.4	0.3, 5.0	3.9	0.5, 7.2	12.2	6.2, 18.2	**0.01**
Breads, and bread rolls, white, mandatorily fortified	4.4	2.4, 7.9	6.6	2.2, 11.1	3.2	0.1, 6.2	2.4	0.3, 5.1	0.28
Fruit juices, commercially prepared	4.1	2.2, 7.3	5.5	1.5, 9.5	7.9	3.1, 12.6	6.8	2.3, 11.4	0.76
Discretionary foods commonly consumed as snacks (%)									
Potato crisps	10.6	7.2, 15.2	16.4	9.9, 22.9	6.5	2.3, 10.6	9.0	3.7, 14.2	**0.04**
Chocolate (plain, unfilled varieties)	9.1	6.2, 13.0	15.5	9.0, 22.0	8.3	3.6, 13.0	6.5	2.1, 10.8	0.07
Soft drinks, cola	6.7	3.5, 12.2	11.3	5.7, 17.0	6.3	1.5, 11.0	1.1	1.0, 3.1	**0.001**
Soft drinks, non-cola	5.0	2.8, 8.8	9.2	4.0, 14.4	6.7	2.4, 10.9	2.6	0.3, 5.5	0.06
Savoury biscuits, wheat based, plain, energy > 1800 kJ per 100 g	4.7	4.7, 10.5	7.1	2.4, 11.9	6.3	2.3, 10.3	11.2	5.5, 16.8	0.38
DGI-CA, Dietary Guideline Index for Children and Adolescents ^29^ Results are presented as weighted proportion (95% CI) ^1^ P-value for differences across tertiles based on Chi-squared test Results are presented as weighted means (95% CI), estimates were determined using multivariate liner regression (adjusted mean), multivariate logistic regression (adjusted proportion), adjusted for age, area-level disadvantage (SEIFA) and reported energy intake to estimated energy requirement (EI: EER). Means and proportions with non-overlapping CI are significantly different form one another (*p* < 0.05)									

Conversely, the proportion of adolescents with a higher diet quality (3rd tertile) consuming discretionary foods as snacks was lower than those with lower diet quality (1st and 2nd tertiles). This difference across levels of diet quality was statistically significant for soft drinks (cola) (*P* = 0.005) and chocolate (plain, unfilled varieties) (*P* = 0.01) among boys, and soft drinks (cola) (*P* = 0.04) and potato crisps (*P* = 0.001) among girls. (Tables 4 and 5) Most adolescents in the top two DGI-CA tertiles, consumed regular whole milk as a beverage without additions at snacks. Furthermore, boys in similar DGI-CA tertiles consumed a higher proportion of regular whole milk at snacks compared to girls. However, a higher proportion of girls consumed whole milk at snacks separately compared to boys. (Supplementary File 2)

To check the robustness of our findings, snack characteristics across DGI-CA were also assessed without excluding EOs with an energy contribution below 210 kJ (Supplementary File 3). The findings indicated that there was no deviation from the distribution of snack characteristics that was observed after excluding EOs with low energy contribution (210 kJ or less). In addition, when examining the top five commonly consumed foods at snacks the excluded EOs were mainly water.

## Discussion

This study aimed to examine the characteristics of snacks among adolescents with different levels of diet quality. The findings indicate that snack ED and the types of food consumed as snacks, but not snack frequency, were associated with diet quality. Snack ED, whether calculated including beverages or not, decreased with increasing diet quality among both boys and girls. Further, the consumption of discretionary foods at snacks decreased with increasing diet quality scores. Whereas the consumption of foods from the five food groups at snacks increased. These findings suggest that snack ED may provide more informative insight into the nutritional profile of snacks than snack frequency alone and underscore the potential of snacks as an opportunity to enhance adolescents’ overall diet quality by promoting the intake of healthy foods at snacks.

Snack frequency has been the most examined characteristic of snacks in relation to diet quality among adolescents. ^11,13,18–20,35–37^ However, studies investigating the association between snack characteristics and diet quality have yielded mixed findings^8^. One possible explanation for this inconsistency could be the use of snack frequency, which is a simplistic measure that does not differentiate snacks on the basis of their nutritional quality but nevertheless demonstrates a positive association with EI^11,38,39^. Findings from the present study found no difference in snack frequency across levels of diet quality scores in both boys and girls. As a result, future studies should refrain from relying solely on snack frequency as the only measure of snacking behaviour in relation to diet quality.

Snack ED is a characteristic that has received limited attention in studies examining adolescent diet quality^13^ A study conducted by Murakami and Livingstone^13^ among children and adolescents aged 4–18 years found an inverse association between snack ED and diet quality as measured by the MDS. Our findings also demonstrated a consistent trend of increasing diet quality with decreasing snack ED. This could be attributed to the fact that diets assessed as having higher diet quality tend to contain low-ED foods^40,41^; therefore, one would expect snacks as part of the diet to include lower ED foods. Considering this, snack ED may be a more useful measure of snacking behaviour than snack frequency, as it provides some insight into the nutritional profile of snacks.

Our findings showed that high proportions of adolescents consumed discretionary foods at snacks (e.g., potato crisps, soft drinks, and savoury biscuits). Moreover, many adolescents also consumed foods from the five food groups at snacks, including milk, apples, and bananas, suggesting that snacks vary in their nutritional quality. Our findings showed that the consumption of foods from the five food groups tended to increase with increasing adolescent diet quality, whereas the opposite was observed for the intake of discretionary foods (higher intake corresponded to lower overall diet quality). A study conducted by Dunford and Popkin^34^ involving U.S. children and adolescents found that the highest energy intake from snacks comes from sugar-sweetened beverages, desserts, and sweets. Conversely, lower energy intake from snacks was observed in fruits and vegetables, meat and fish, salty snacks, and dairy products. These findings provide insight into the potential contribution of snacks to the overall diet quality observed among U.S. children and adolescents^2^. Based on these findings, it can be inferred that public health campaigns targeting adolescent diet quality should concentrate on substituting the consumption of commonly consumed discretionary foods such as potato crisps, chocolate, soft drinks, and savoury biscuits with commonly consumed foods from the five food groups, such as fruits and milk, at snacks. Apple was the most consumed food from the five food groups at snack, with nearly third of those in the highest tertile and around one in five adolescents overall. Given that previous research has revealed that adolescents are in need of more peer support and peer role modelling to promote health eating^42^, the notably higher consumption of apples at snacks could serve as an initial starting point for peer support and peer role modelling interventions. Hence, increasing healthier food choices at snacks, especially where peer support and role modelling are desired could be beneficial.

In the present study, beverages were widely consumed at snacks, particularly soft drinks (both cola and non-cola) and milk. Boys and girls with the lowest diet quality (bottom two tertiles) had a much higher consumption of soft drinks than those with a higher diet quality (top tertile). This high intake of soft drinks among adolescents with low diet quality may put them at an increased risk of obesity, as supported by a previous systematic review and meta-analysis by Malik, et al.^43^, which revealed a link between sugar-sweetened beverages, weight gain, and obesity. Interventions designed to reduce the consumption of soft drinks at snacks may not only enhance the diet quality of those with poor diet quality but also complement efforts to reduce obesity prevalence in this age group.

Despite the importance of dairy in promoting the dietary intake of calcium, protein, iodine, vitamin A, vitamin D, and zinc, the 2011–2012 NNPAS findings revealed suboptimal dairy consumption among adolescents^44^. This was reflected in the inadequate calcium intake in this age group, particularly among girls^45^. Surprisingly, this study found that milk was among the top five snack foods consumed by both boys and girls. Similarly, a study conducted among U.S. children and adolescents showed that milk/dairy is among the commonly consumed foods at snacks^46^. However, the proportion of adolescents who consume milk at snacks remains relatively low. This observation underscores the potential of snacks as an opportunity to promote the likelihood of adolescents meeting the daily recommended servings of dairy foods and micronutrients, such as calcium.

There was a modest difference in the consumption of full-fat milk among boys across tertiles but not among girls. The highest proportion of adolescent boys who consumed full-fat milk as a snack was observed in the 2nd tertile. However, no clear trend was observed in the proportion of adolescents consuming full-fat milk across tertiles of DGI-CA. Milk has been favourably scored in the DGI-CA component “a variety of nutritious foods,” but extra points have been given to dairy and/or alternatives with mostly reduced fat^29^. Adolescents would have scored higher on the DGI-CA if milk consumed at snack was reduced in fat. Moreover, nearly all milks consumed as a beverage were unflavoured which has been previously associated with a lower proportion of daily energy from free sugar than flavoured milk drinkers, other milk drinkers (e.g., smoothies, milkshakes), non-drinkers of milk (e.g., only consume milk as an addition to cereal or other foods)^44^.

Adolescents are more autonomous in their food choices than younger children and may be consuming snacks purchased for them or by them^47,48^. Adolescents’ food choices are influenced by the familiarity, availability, price, nutritiousness, taste, and convenience of foods and may also be related to social influences^49^. Fruit and milk are among the commonly consumed foods at snacks, possibly because of their familiarity and convenience for on-the-go consumption. A research project conducted among Danish adolescents^50,51^ explored their snacking behaviour and aimed to develop and assess novel, healthier snacking options. The study revealed the key aspects that adolescents sought in these new snack alternatives: a mix of high and low ED, a balance between healthy and indulgent choices, incorporation of items such as berries and meat, diverse taste experiences, and various textures (spanning sweet and savoury; juicy, crisp, and soft). Additionally, the snacks were ready-to-eat, packaged in resealable formats, and suitable for on-the-go consumption. Building on these insights, a range of snacks were introduced, including apple wedges, pineapple mini sticks, mini cucumbers, orange and white mini carrots, mild salsa dips, chopped unsalted peanuts, and bacon crisps. Subsequent testing indicated a favourable acceptance of these new options for adolescents^50,51^. This demonstrates that the development of healthy snacks tailored to adolescents’ needs and making them available and accessible to adolescents can play a role in promoting healthier snacking habits and ultimately contribute to improved overall diet quality.

The present findings should be interpreted with consideration of the following limitations. The survey was conducted between 2011 and 2012, so it may not reflect current snaking habits among Australian adolescents’. However, it is the most recent data available. With the next survey data expected in 2025, this study’s findings can help track changes in adolescent snacking habits over time^15,37^. The findings might also be relevant to other high-income countries like the U.S. and UK, which have reported similar adolescent snacking patterns associated with lower diet quality^11,15,37,52^. Additionally, more recent studies from the U.S. indicate that snacking remains prevalent among adolescents and that these snacks are predominantly unhealthy^15,37^. The present study focused on examining the characteristics and nutritional quality of snacks, including snack frequency, snack energy density, and commonly consumed foods during snacks among adolescents with different levels of diet quality. Future research could explore other snack characteristics, such as macronutrient and micronutrient content, and consider including other eating occasions (i.e. meals) alongside snacks to provide a comprehensive understanding of the relationship between snacks and diet quality. Snack characteristics may vary between weekdays and weekends, as well as in their relationship with diet quality. Dutch, et al.^53^ showed that weekends were characterized by higher discretionary food intake, which made up a larger portion of adolescents’ snacks, and lower diet quality compared to weekdays. However, the NNPAS 2011–2012 underrepresented Sundays, which may result in dietary intakes not being fully representative of weekends^54^. Future studies should explore the relationship between snack characteristics and diet quality on weekdays versus weekends. The use of a single 24-hour dietary recall to examine the characteristics of snacks among adolescents is unlikely to capture day-to-day variation in snacking behaviour. However, a study using the NNPAS 2011-12 did not observe significant differences in snack frequency or snack contribution to energy intake between children and adolescents who completed one or both days of recall^21^. Interviewing adolescents aged 12–14 years with a proxy present may lead to recall and reporting bias, especially for unhealthy foods. This could result in underreporting of snack intake, particularly discretionary foods. Nonetheless, more recall days may be needed to better understand within-person variability in snack characteristics. Although our models adjusted for potential energy intake misreporting, the calculations for EI: EER assumed that all adolescents belonged to the low-active physical activity group, despite the variability of level of physical activity among adolescents^55^. However, the majority of adolescents did not meet the daily minimum of 60 min of moderate- to vigorous-intensity physical activity on most or all days; hence, assigning low-active physical activity values might have minimum impact^55^. Snacks were identified based on participant-identification of EOs which involves subjectivity in participants’ allocation of an EO as a meal or snack. However, a study using the NNPAS 2011-12 have showed a high agreement in snack frequency between participant-identified and time-of-day definition approaches for identifying EO^21^. Preliminary evidence suggests that, among adolescents, the contribution of snacks to EI and snack consumption vary across consumption location (at home and away from home) and where snacks were sourced (e.g., grocery store, convenient store, vending machine or from someone else/gift)^56,57^. Although the source of snacks and location of consumption may play a role in the relationship between snack characteristics and overall diet quality, these factors could not be considered in this study since they were not measured in the NNPAS. Future surveys should include objective or more detailed measures of physical activity for a more accurate estimation of the EER. In addition, future studies may also explore the influence of snack source and location of consumption on adolescent’s snack characteristics and diet quality. The strengths of the present study include the use of an evidence-based approach to define snacks and the evaluation of multiple characteristics of snacks (snack frequency and snack ED). Dietary data were collected from a nationally representative sample of Australian adolescents during all months of the year, which may capture the possible variability in snacking and diet quality on school and non-school days, and across seasons among adolescents.

## Conclusions

In a nationally representative cross-sectional sample of Australian adolescents, higher snack ED was associated with lower diet quality. Among adolescents with higher diet quality, the proportion of snacks comprising discretionary foods was lower, whereas the proportion of snacks comprising foods from the five food groups was higher. No associations between snack frequency and diet quality were observed. These findings suggest snack ED may offer more insight into snack nutritional quality than snack frequency alone; however, further research examining differences in snack characteristics by diet quality over multiple dietary intake days and in other adolescent populations are needed to support these findings. Future studies research should also consider investigating multiple snack characteristics, including frequency, ED, and foods commonly consumed at snacks, to gain a comprehensive understanding of snacks in relation to diet quality. Finally, strategies aimed at enhancing the desirability and accessibility of foods from the five food groups for consumption as snacks warrant investigation in future research.

## Electronic supplementary material

Below is the link to the electronic supplementary material.

Supplementary Material 1

Supplementary Material 2

Supplementary Material 3

Supplementary Material 4

## Data Availability

Data are available on request from the Australian Bureau of Statistics from https://www.abs.gov.au/statistics/microdata-tablebuilder. The research team does not have the permission to release the data to third parties.
