# Supplementary material for: Comparison of snack characteristics by diet quality findings from a nationally representative study of Australian adolescents
Source: Sci Rep. 2024 Oct 10;14:23663. doi: 10.1038/s41598-024-75386-1 (PMC11466951; doi:10.1038/s41598-024-75386-1)
Supplement: Supplementary file 1 — Supplementary Material 1 [file 41598_2024_75386_MOESM1_ESM.docx]

Supplementary File 3. Characteristics of snacks (without excluding snacks <210 kJ) across DGI-CA categories among adolescent boys aged 12 -18 year from the National Nutrition and Physical Activity Survey 2011-12.

|  | **Tertile of Diet quality (DGI-CA)** | | |  |
| --- | --- | --- | --- | --- |
|  | 1^st^ tertile (n= 160) | 2^nd^ tertile (n= 146) | 3^rd^ tertile (n= 181) | P value |
| **Characteristics** | Mean | Mean | Mean |  |
| **Boys** |  |  |  |  |
| Snack frequency | 5.4 | 5.8 | 5.7 | 0.0690 |
| ED of snacks including beverages (kJ/g) | 3.8 | 3.4 | 3.0 | 0.0000 |
| ED snacks without beverages (kJ/g) | 4.4 | 3.9 | 3.3 | 0.0001 |
| **Girls** |  |  |  |  |
| Snack frequency | 5.2 | 5.9 | 5.8 | 0.0078 |
| ED of snacks including beverages (kJ/g) | 3.6 | 3.0 | 2.7 | 0.0000 |
| ED snacks without beverages (kJ/g) | 4.2 | 3.2 | 3.0 | 0.0000 |

Commonly consumed foods at snack

1. Domestic water (including tap, tank/rain water)
2. Milk, cow, fluid, regular whole, full fat
3. Breads, and bread rolls, white, mandatorily fortified
4. Sugar
5. Soft drinks, cola
