# Supplementary material for: Comparison of snack characteristics by diet quality findings from a nationally representative study of Australian adolescents
Source: Sci Rep. 2024 Oct 10;14:23663. doi: 10.1038/s41598-024-75386-1 (PMC11466951; doi:10.1038/s41598-024-75386-1)
Supplement: Supplementary file 2 — Supplementary Material 2 [file 41598_2024_75386_MOESM2_ESM.docx]

Supplementary File 1 Participant flowchart for inclusion in the analysis of adolescent snacking
