# Supplementary material for: Comparison of snack characteristics by diet quality findings from a nationally representative study of Australian adolescents
Source: Sci Rep. 2024 Oct 10;14:23663. doi: 10.1038/s41598-024-75386-1 (PMC11466951; doi:10.1038/s41598-024-75386-1)
Supplement: Supplementary file 3 — Supplementary Material 3 [file 41598_2024_75386_MOESM3_ESM.docx]

Supplementary File 2. Proportion of full fat milk according to combination food code among adolescents between 12 -18 year from the National Nutrition and Physical Activity Survey 2011-12.

|  | **Full fat milk consumed as a snack** | | |
| --- | --- | --- | --- |
| **SEX** | **Consumed separately** | **Beverage with additions** | **Cereal with additions** |
| **Male** |  |  |  |
| 1^st^ tertile | 42% | 53% | 5% |
| 2^nd^ tertile | 68% | 31% | 1% |
| 3^rd^ tertile | 67% | 23% | 10% |
| **Female** |  |  |  |
| 1^st^ tertile | 64% | 36% |  |
| 2^nd^ tertile | 75% | 25% |  |
| 3^rd^ tertile | 71% | 29% |  |

Table 2 The top foods consumed as snack among male adolescents between 12 -18 year from the National Nutrition and Physical Activity Survey 2011-12.

| **Tertiles** | **Proportion** | **95% CI** |
| --- | --- | --- |
| **1st tertile** |  |  |
| Soft drinks, cola | 20.0% | (12.9, 29.7) |
| Potato crisps | 15.9% | (10.6, 23.2) |
| Soft drinks, non-cola | 13.1% | (7.7, 21.4) |
| Chocolate (plain, unfilled varieties) | 8.0% | (3.9, 15.5) |
| Cordials, made from concentrate | 12.5% | (6.9, 21.5) |
| **2nd tertile** |  |  |
| Apples | 26.8% | (16.3, 40.7) |
| Potato crisps | 19.4% | (12.1, 29.7) |
| Milk, cow, fluid, regular whole, full fat | 23.1% | (13.7, 36.1) |
| Lollies and other confectionery, sugar sweetened | 16.2% | (9.4, 26.6) |
| Chocolate (plain, unfilled varieties) | 10.5% | (5.0, 20.7) |
| **3rd tertile** |  |  |
| Apples | 32.2% | (23.4, 42.6) |
| Bananas | 14.0% | (8.6, 22.0) |
| Milk, cow, fluid, regular whole, full fat | 13.0% | (6.9, 23.0) |
| Potato crisps | 13.9% | (8.0, 23.0) |
| Milk, cow, fluid, reduced fat, <2 g/100g | 6.5% | (3.4, 12.2) |

Table 3 The top foods consumed as snack among female adolescents between 12 -18 year from the National Nutrition and Physical Activity Survey 2011-12.NNPAS.

| **Tartiles** | **Proportion** | **95% CI** |
| --- | --- | --- |
| **1st tertile** |  |  |
| Soft drinks, cola | 10.2% | (4.9, 20.2) |
| Potato crisps | 16.8% | (9.5, 28.1) |
| Soft drinks, non-cola | 5.8% | (2.7, 12.1) |
| Chocolate (plain, unfilled varieties) | 10.2% | (4.7, 20.6) |
| Cordials, made from concentrate | 5.8% | (2.2, 14.0) |
| **2nd tertile** |  |  |
| Apples | 9.8% | (4.5, 19.7) |
| Potato crisps | 10.0% | (4.7, 19.8) |
| Milk, cow, fluid, regular whole, full fat | 7.1% | (2.9, 16.1) |
| Lollies and other confectionery, sugar sweetened | 8.2% | (3.1, 19.7) |
| Chocolate (plain, unfilled varieties) | 11.7% | (6.6, 19.7) |
| **3rd tertile** |  |  |
| Apples | 29.6% | (20.9, 40.0) |
| Bananas | 16.4% | (8.9, 28.5) |
| Milk, cow, fluid, regular whole, full fat | 5.0% | (1.7, 13.4) |
| Potato crisps | 4.9% | (2.3, 10.3) |
| Milk, cow, fluid, reduced fat, <2 g/100g | 12.3% | (6.9, 21.0) |
