# Supplementary material for: Comparison of snack characteristics by diet quality findings from a nationally representative study of Australian adolescents
Source: Sci Rep. 2024 Oct 10;14:23663. doi: 10.1038/s41598-024-75386-1 (PMC11466951; doi:10.1038/s41598-024-75386-1)
Supplement: Supplementary file 4 — Supplementary Material 4 [file 41598_2024_75386_MOESM4_ESM.docx]

List of abbreviations

ABS Australian Bureau of Statistics

CI Confidence intervals

DGI-CA Dietary Guideline Index for Children and Adolescents ()

DQI-A Diet Quality Index for Adolescents

DRI Dietary Reference Intake

DUHREC Deakin University Human Research Ethics Committee

ED Energy density

EER Estimated energy requirement

EI Energy intake

EO Eating occasions

FSANZ Food Standards Australia New Zealand

IPAN Institute for Physical Activity and Nutrition

MDS Mediterranean Diet Score

NNPAS National Nutrition and Physical Activity Survey

SEIFA Area-level disadvantage

USDA U.S. Department of Agriculture
